# Acceptance and Commitment Therapy for Addressing Chronic Cancer Pain: A Narrative Review with Considerations for Utilization in Primary Care

**DOI:** 10.3390/healthcare14193232

**Published:** 2026-10-01

**Authors:** Nina H. Russin, Kevin Pardon, Jennifer Sander, Ronald O’Donnell, Matthew P. Martin

**Affiliations:** 1 College of Health Solutions, Arizona State University, Health North, 550 N. 3rd St., Phoenix, AZ 85004, USA; 2 Arizona State University Library, Arizona State University, 411 N Central Ave, Suite L1-61, Phoenix, AZ 85004, USA; 3 Sandstone Counseling & Consultation, LLC, Peoria, AZ 85383, USA

**Keywords:** cancer pain, acceptance and commitment therapy, integrated behavioral health, primary care, low-and-middle-income countries

## Abstract

Background: Chronic pain is common among cancer survivors and may reflect interacting physical, psychological, social, and existential factors. Acceptance and Commitment Therapy (ACT) is a behavioral intervention that emphasizes psychological flexibility, acceptance, present-moment awareness, and values-guided action. As cancer survivorship care increasingly involves primary care, scalable behavioral approaches may be needed to complement medical pain management, particularly where specialty behavioral health resources are limited. Methods: This narrative review examined evidence for ACT in chronic cancer-related pain and its potential applicability to primary care and resource-constrained healthcare settings. Searches were conducted in PubMed, PsycINFO, and CINAHL Ultimate, supplemented by Google Scholar and the Association for Contextual Behavioral Science database. Fifteen studies met criteria for inclusion. Results: ACT showed promising effects on outcomes including psychological flexibility, pain acceptance, emotional distress, quality of life, pain coping, and pain-related functioning, whereas findings for pain intensity were less consistent. The evidence was limited by heterogeneous study designs and populations and generally small experimental samples. None of the included studies directly evaluated ACT for cancer-related pain in primary care, although studies in other chronic pain and mental health populations provide indirect support for the feasibility of ACT in primary care and community settings. Discussion: Although direct evidence for the utilization of ACT in primary care is lacking, studies on ACT interventions for chronic pain and mental illness in these settings suggest that brief ACT interventions for cancer pain may help to address the need for behavioral health support. Future research should include pragmatic trials to determine whether brief ACT-informed interventions can be feasibly integrated into multidisciplinary primary care teams and delivered through digital or hybrid models.

## 1. Introduction

Pain is one of the most feared consequences of a cancer diagnosis, with life-altering implications. It is estimated that between 45% and 50% of cancer survivors experience pain [1], with prevalence reaching up to 76% of individuals with advanced cancer [2]. Dame Cicely Saunders, the British nurse and social worker credited with starting the hospice care movement, described the cancer experience as “total pain” due to its multi-dimensional character, including physical, existential, psychological, social, and spiritual components [3,4]. Adding to its complexity is the changing nature of cancer pain over time, reflecting physiological alterations such as tumor invasion and organ compression, negative side effects of certain chemotherapies and radiation treatments, subjective fear of a life-limiting illness, stigma, and social isolation. Comorbidities including stress, anxiety, depression, sleep disturbance, and fatigue are not uncommon [2].

### 1.1. Cancer Pain in Low-and-Middle-Income Countries

The highest burden of cancer pain occurs in low-and-middle-income countries (LMICs), where patients often present for treatment at more advanced stages of cancer and healthcare resources are scarce [1,2]. Emerging research suggests that cognitive behavioral interventions might help to address this gap in care. Cultural norms in Asia, where families make decisions collectively regarding sharing a cancer diagnosis with the patient, are vastly different than they are in the US, where patient autonomy is highly valued [5]. Withholding information from cancer patients is commonplace in many Asian countries, including China, Japan, and Singapore, due to concerns that this might cause additional harm to the patient (e.g., emotional distress) [6]. These cultural factors will be important to consider in adapting cognitive behavioral therapies for patient populations in eastern and western cultures.

Cancer-related stigma presents additional barriers in Asia, as reported in a multi-site study of patients with advanced cancer from 11 hospitals in eight countries in Asia [7]. Patients reported not being part of decision making, not being treated with dignity, lack of adequate consultation time with providers, and not receiving clear information regarding their diagnosis and treatment options. In a second study among 1358 patients with advanced cancer in Bangladesh, China, India, the Philippines, Sri Lanka, and Vietnam, 60% stated that they occasionally perceived stigma, leading to negative mental health outcomes including anxiety and depression [8]. These barriers suggest that many cancer survivors globally are experiencing unnecessary suffering, pointing to a need for more readily available pain treatment strategies.

### 1.2. Acceptance and Commitment Therapy for Cancer Pain

Recent research suggests benefits from mindfulness-based therapies for cancer pain [9,10]. Acceptance and Commitment Therapy (ACT) is considered a “third wave” cognitive behavioral approach that combines meditation practices such as deep breathing and present-moment awareness with more traditional CBT strategies [11]. ACT focuses on six core processes: present-moment awareness, dimensions of self, defusion, acceptance, connecting with values, and committed action, all described below in a separate section [12]. As such, it combines elements of other mindfulness and meditation practices (present-moment awareness, acceptance) and cognitive behavioral approaches (committed action) with an overall focus on connecting with one’s long-term values, which may help one to navigate through challenging periods in life. Since much of cancer-related pain is chronic (symptoms lasting three months or more), the focus on long-term values can be an important motivator for engaging in meaningful life activities including physical activity, good nutrition, and socialization.

Key to ACT is the concept of psychological flexibility, which proposes that “pain is a natural consequence of living, but that people suffer unnecessarily when their level of psychological rigidity prevents them from adapting to internal or external concepts” (p. 64, [12]). Psychological inflexibility can result from several maladaptive processes, including inflexible attention (often focused on the past or future versus the present moment), attachment to a conceptualized self, and avoidance of values-based committed action. While cognitive reappraisal is central to both CBT and ACT, the nuance is slightly different. CBT is structured around specific maladaptive thought patterns such as fortune telling, black-and-white thinking, catastrophizing and mind reading, whereas ACT adds specific focus on the *context* within which these thought patterns occur. For example, fear of pain in the context of cancer survivorship and the possibility of recurrence is quite different from phobia, which is an irrational fear of pain. Therefore, an ACT therapist might encourage a client to meaningfully engage with that experience as it occurs in the present moment, rather than avoiding that experience (which could potentially amplify the fear).

### 1.3. Six Core Processes of Acceptance and Commitment Therapy

The six core processes are divided into two categories: commitment and behavioral activation, and mindfulness and acceptance processes [12]. Acceptance is particularly focused on the emotional aspects of the human experience [12], making it particularly relevant to self-management of cancer pain. Specifically, ACT teaches clients to explore their personal experiences (including pain), rather than avoiding them [12]. Reconsidering Dame Cicely Saunders’ description of “total pain,” individuals faced with both existential pain (related to the diagnosis of a life-limiting illness) and psychological pain (emotional distress, stress, anxiety, and depression) may become their pain [12]. ACT suggests that a contextual shift from “being in pain” to “observing pain” can give clients the opportunity to move forward to a more values-driven life [12]. The concept of defusion is also relevant here. Fusion, in the ACT context, refers to melding verbal and cognitive processes with direct experience, so the individual is unable to separate these constructs. In other words, pain is not only chronic, but also persistent within all current experience. It dictates behavior, in particular, “experiential avoidance” [12]. ACT challenges patients to experience the present with an attitude of openness and curiosity, which opens the door for positive experiences of human connection and joy. This is related to the mindfulness concept of present-moment awareness: focusing on the present and putting disruptive thoughts to the side.

### 1.4. Acceptance and Commitment Therapy in Primary Care

As advances in cancer detection and treatment continue to improve survival, an increasing proportion of cancer survivors transition from oncology to primary care for long-term follow-up and management of persistent symptoms. Contemporary survivorship models emphasize greater collaboration between oncology and primary care, with approximately two-thirds of cancer survivors receiving most of their ongoing healthcare from primary care physicians [13]. Systematic reviews have further demonstrated that shared-care and primary care-led survivorship models can achieve outcomes comparable to specialist-led follow-up while improving continuity of care and potentially reducing healthcare costs [14,15].

However, implementation challenges remain. Primary care physicians consistently report limited confidence in managing chronic cancer-related pain, citing insufficient education, limited clinical guidance, fragmented communication with oncology teams, uncertainty regarding long-term opioid management, and time constraints during routine office visits [16,17,18]. Clinicians across oncology, palliative care, and primary care have similarly described chronic cancer pain management as having “no clear home,” resulting in uncertainty regarding responsibility for long-term pain management following completion of cancer treatment [19,20]. These findings highlight the need for practical, evidence-informed, and scalable approaches that can complement existing medical management and be adapted to resource-constrained primary care settings. Cognitive behavioral interventions fill an important gap, in that they *integrate* structured approaches to the psychological aspects of cancer pain, which may include stress, anxiety, depression, and resulting decrements in quality of life.

### 1.5. Cancer Survivorship in Asian Nations

In some Asian nations (e.g., China), cancer care does not occur in primary care, but only in tertiary centers specializing in oncology [21]. “Healthy China 2030” proposes that medical education include more oncology-specific care for general practitioners, but this does not necessarily include behavioral health. While the Chinese government supports integrated behavioral health in primary care, routine implementation remains limited due to severe time constraints that general practitioners must adhere to in order to treat very high volumes of patients, as well as a lack of behavioral health training [22].

### 1.6. Brief ACT-Informed Conversations

These implementation challenges highlight the need for brief behavioral interventions that can complement medical management of chronic cancer-related pain while remaining feasible within routine primary care. ACT possesses several characteristics that align with these needs. Accordingly, the objective of this narrative review is to synthesize the current evidence regarding ACT for chronic cancer-related pain and to consider whether its clinical outcomes and delivery models support evidence-informed integration into primary care. As part of this examination, we also address the use of paraprofessionals such as community health workers to extend patient conversations beyond the physician encounter, in order to help cancer survivors improve their self-management of cancer-related pain.

The literature reviewed here focuses primarily on psychological and functional dimensions of cancer-related pain rather than its underlying structural causes, consistent with ACT’s emphasis on psychological flexibility, acceptance, and values-guided behavior. These processes may be particularly relevant to cancer survivors for whom pain is intertwined with emotional distress, fear of recurrence or disease progression, and disruption of meaningful activities. Accordingly, this review examines the evidence for ACT in chronic cancer-related pain and considers its implications for brief, ACT-informed approaches that could complement medical management within the time and resource constraints of primary care.

## 2. Methods

A narrative review was deliberately selected to accommodate the broad scope of the research objectives and the heterogeneity and emerging nature of the literature on this topic, as described above. Our goal was not only to evaluate potential gaps in the literature but also to suggest a path forward for primary care clinicians faced with increasing numbers of cancer survivors managing cancer-related pain. To address historical critiques regarding the reporting in narrative reviews, the PRISMA principles were adapted to ensure methodological transparency and reporting rigor throughout the search and selection process.

Literature search strategies were developed in collaboration between the first author (NR), a Doctor of Behavioral Health, and the second author (KP), a health sciences librarian, to minimize potential selection bias. A concept map was developed to identify primary search domains, including primary concepts of “cancer,” “pain,” and “Acceptance and Commitment Therapy,” while secondary concepts included “total cancer pain,” “ACT in low-and-middle-income countries,” “ACT in primary care,” and “mHealth technology and ACT availability,” particularly for individuals with advanced cancer, limited transportation options, or living a long distance from healthcare facilities. The final author (MM) reviewed the selected studies for relevance.

Preliminary test searches incorporating both the primary and secondary concepts were conducted. To maximize search sensitivity, final search strategies were subsequently developed and focused only on the primary concepts. Searches were completed in the PubMed, PsycINFO, and CINAHL Ultimate databases as follows:

PubMed (85 results, 28 July 2026):

Search Thread: (ACT [Title/Abstract] OR “Acceptance and Commitment Therapy” [MeSH Terms]) AND (“chronic pain” [MeSH Terms] OR chronic pain [Title/Abstract]) AND (“Neoplasms” [MeSH] OR cancer [Title/Abstract] OR neoplasm [Title/Abstract] OR tumor [Title/Abstract] OR carcinoma [Title/Abstract] OR oncolog* [Title/Abstract] OR maligna* [Title/Abstract])

PsycINFO (68 results, 26 July 2026):

Search Thread: (MAINSUBJECT.EXACT (“Acceptance and Commitment Therapy”) OR ACT OR “Acceptance and Commitment Therapy”) AND (MAINSUBJECT.EXACT (“Chronic Pain”) OR chronic pain) AND (MAINSUBJECT.EXACT.EXPLODE (“Neoplasms”) OR cancer OR neoplasm OR tumor OR carcinoma OR oncology OR malignancy)

CINAHL Ultimate (17 results, 28 July 2026).

Search Thread: XB (MH “Acceptance and Commitment Therapy” OR ACT OR “acceptance and commitment therapy”) AND XB (MH “Chronic Pain” OR chronic pain) AND XB (MH “Neoplasms” OR neoplasm OR tumor OR carcinoma OR oncology OR malignancy)

The primary database searches yielded 170 results, of which 26 were duplicates. A targeted supplementary search of “acceptance and commitment therapy” AND cancer AND chronic pain was conducted in Google Scholar. The first three pages of results (30) were examined to minimize the risk of omitting any key literature that may not have been retrieved in the primary database searches. Lastly, a manual search of the Association for Contextual Behavioral Science (ACBS) database of ACT interventions in LMICs was conducted.

Inclusion criteria: Eligible articles were peer-reviewed, English-language publications that evaluated, described, or synthesized ACT interventions relevant to chronic cancer-related pain, including its psychological and functional dimensions. Primary studies and secondary reviews were eligible. Studies examining patient engagement with ACT, including barriers to participation and strategies to improve treatment retention, were also included when relevant to the review objectives. The review primarily focused on literature published within the previous 10 years; older studies were included when they provided directly relevant evidence not adequately represented in the more recent literature.

Exclusion criteria: Gray literature, including dissertations, conference proceedings, and other non-peer-reviewed publications, was excluded. Articles were also excluded if they addressed cancer-related pain without an ACT component, ACT without relevance to cancer or cancer-related pain, or chronic pain populations for which cancer-specific findings could not be distinguished.

**Figure 1:**
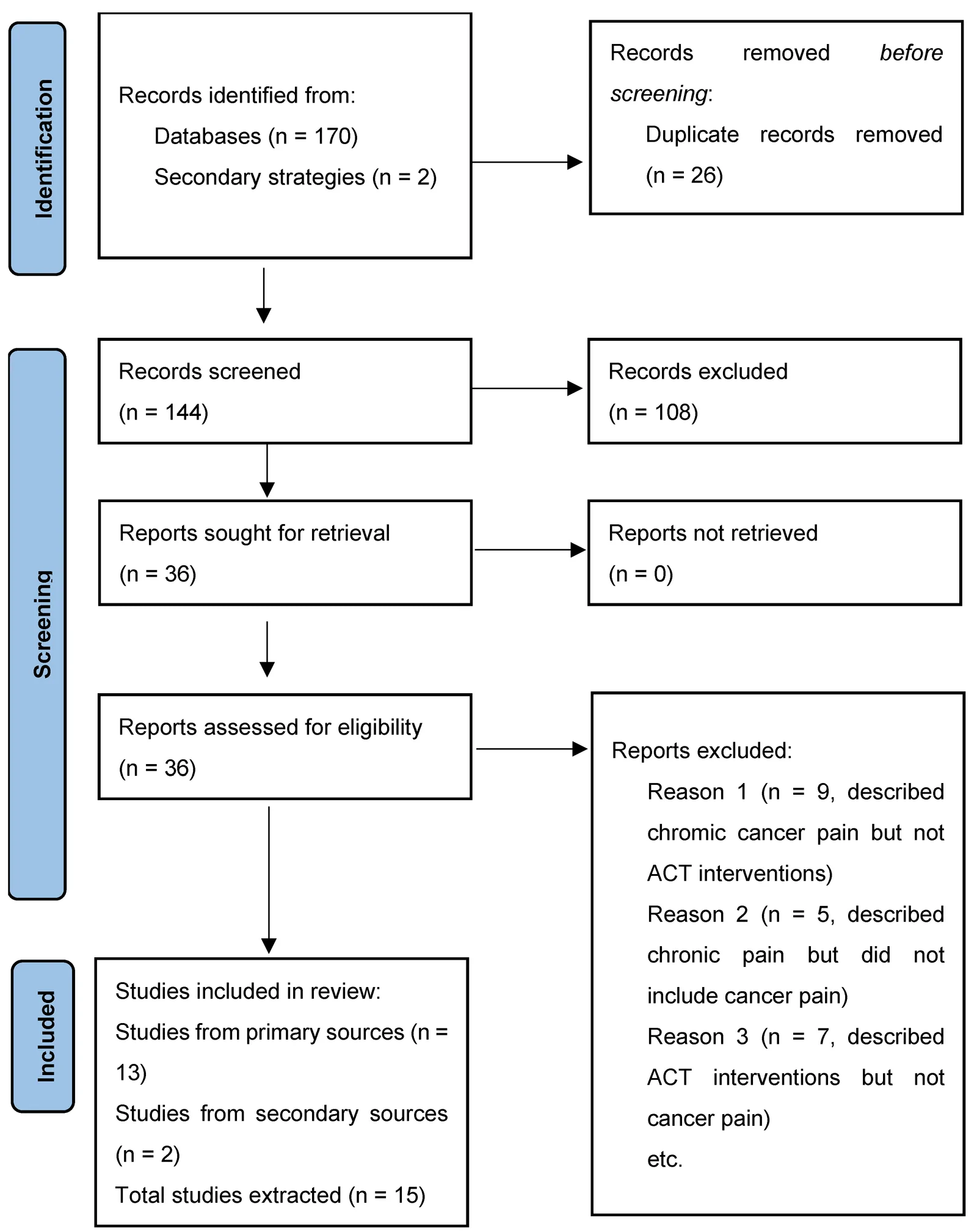
PRISMA flow diagram.

Of the 170 articles identified through the primary database searches, 26 were duplicates. These were removed, and 144 records were screened against the inclusion and exclusion criteria. Of these, 36 articles underwent full-text screening, resulting in 13 studies selected for inclusion. Two additional articles were identified through the secondary search strategies, one from Google Scholar and one from the ACBS database, bringing the final total to 15 included studies in this narrative review. A PRISMA flow diagram outlines the study identification, screening, and selection process for primary and supplementary searches (Figure 1).

Given the narrative purpose of this review and the substantial heterogeneity of the included evidence, which encompassed primary quantitative and qualitative studies as well as systematic, scoping, and narrative reviews, a formal risk-of-bias or methodological quality assessment was not undertaken. The review was intended to characterize and interpret the emerging evidence rather than estimate pooled treatment effects or grade certainty of evidence. Methodological limitations of individual studies and the overall evidence base were instead considered narratively when interpreting the findings.

## 3. Results

Table 1 summarizes the extracted studies by country of origin, participant demographics, and type and stage of cancer, while Table 2 describes study designs, type(s) of pain targeted, and study results.

Of the 15 studies selected for formal review, ten specifically addressed the use of ACT for cancer pain [24,25,27,28,30,32,33,35,36,37], while two included additional mindfulness interventions [26,31], and one included other chronic health conditions [29]. One article specifically addressed the issue of dropout in ACT therapy [34], which is particularly relevant to its utilization in patients living with complex chronic conditions. In addition, a quantitative study focused on anxiety and quality of life in stomach cancer patients [23]. While pain was not listed specifically as an outcome, we included this study because the authors made a clear connection between these psychological constructs and the physical consequences of cancer (e.g., chronic pain). Two of the articles [36,37] derived from a single source and reported on patient engagement in development and results for a qualitative pilot study within a larger randomized controlled trial.

Study designs were heterogeneous in nature, including systematic reviews, a scoping review, a narrative review, and experimental studies. Experimental studies, with one exception, involved small sample sizes, which limits their generalizability. However, these smaller studies included rich qualitative data from patient interviews, which was directly relevant to the efficacy of utilizing ACT for cancer pain.

Secondary sources [25,26,27,29,31,32,35] introduce the possibility that some of the experimental results cited here were counted more than once. The first author carefully reviewed studies included in these secondary reports to ensure that there was no overlap. This involved reviewing tables within those articles for information about study inclusion and, in one case where the article lacked such tables, reviewing the article’s reference list. In cases where studies included types of chronic pain in addition to cancer pain, the authors only included results specific to cancer pain.

### 3.1. Preliminary Findings

Consistent findings across experimental studies included small sample sizes [23,24,28,30,33,36] and heterogeneity among both study populations and study designs, which makes direct comparison of outcomes difficult. Given these limitations, ACT interventions were found to be culturally acceptable [33], with positive small-to-moderate effects on pain coping [28,30] and pain acceptance [33] and more limited evidence for pain severity and interference [24,25,26,35].

Studies originated from North America, Western Europe, Australia, the Middle East, and Asia, with researchers implementing ACT interventions in surgical units, oncology practices, palliative care, and remote (digital/telehealth) delivery. While the ACT framework was initially developed for use by clinical psychologists, it is versatile enough to translate well to other types of patient encounters. This is the driving force behind what ACT founders call “contextual behavioral science,” which encourages clinicians to adapt ACT to fit the needs of resource-constrained healthcare systems [12]. A consideration is the time requirement for patients receiving ACT, which is significant and can be burdensome for individuals receiving various types of therapy from multiple providers. In some cases, additional behavioral strategies such as MI have been used to improve retention in treatment [34].

### 3.2. Use of ACT in Primary Care

None of the studies in this review took place in primary care or community settings. Therefore, we cannot cite any direct evidence supporting the use of ACT in primary care to address the psychological dimensions of cancer-related pain. However, ACT interventions in primary care have been studied for chronic pain [38,39] and mental illness [40]. In addition, there is a growing body of literature on the use of ACT in community health settings in low-and-middle-income countries (LMICs) [41,42,43]. While not directly applicable, these studies support the feasibility of implementing ACT in environments that have not traditionally included mental health care. We will elaborate on this concept in the discussion section.

### 3.3. The Importance of Patient Engagement

Patient engagement was essential to ensure intervention completion [34,36]. The ACT process is not a ‘quick fix’; it requires a significant commitment in time and energy. In addition to dealing with cancer-related fatigue and difficulty concentrating (e.g., chemo brain), participants have to fit time for the ACT intervention within other scheduled provider visits, as well as work and family obligations [33,34]. Helping patients to understand the value of ACT within their personal context can improve patient adherence, as noted in the results of a controlled non-randomized cohort study that utilized brief motivational interviewing to improve patient adherence to ACT [34]. The MI session focused on patients’ personal values, autonomy, and how the group ACT intervention could help participants move forward towards long-term values. MI interviewers also acknowledged difficulties that might be encountered by patients, such as time and scheduling. While motivational interviewing added an additional session, the odds of completing the ACT intervention were five times higher than in the control group that did not receive MI.

In a second study, researchers recruited 12 cancer survivors with chronic peripheral neuropathy from chemotherapy to participate in the development of an online ACT intervention [36]. ACT processes that were particularly meaningful to these individuals included acceptance of their pain and learning to live a meaningful life despite it, mindfulness exercises, and cognitive defusion. They valued peer support but also appreciated the flexibility of a digital intervention. Because of chronic fatigue, they emphasized that exercises should be short. They all agreed on the importance of recognizing cancer as a life-limiting illness and felt that it was important to implement ACT at hospitals and on patient websites.

### 3.4. Intersection of Pain and Emotion

The International Association for the Study of Pain (IASP) defines pain as “an unpleasant sensory and emotional experience associated with, or resembling that associated with, actual or potential tissue damage” [44]. Despite this, many cancer survivors consider their physical cancer-related pain to be unrelated to mood. Several studies reported concurrent improvements in both physical and psychological pain [24,25,28,30,33]. In a Malaysian study of breast cancer survivors, participants reported increased awareness of the interaction of pain and emotion, and better psychological flexibility to engage in personally meaningful activities [33]. A second study on breast cancer survivors in Iran reported significantly reduced mean scores in depression, along with increased pain acceptance and psychological flexibility [28]. A randomized pilot feasibility study on 54 breast cancer survivors found that a single session of ACT resulted in small within-group improvements in pain and anxiety three months after surgery [30]. Finally, a pilot randomized trial of advanced gastrointestinal cancer survivors reported moderate within-group reductions in pain severity and interference, concurrent with reductions in depression and sleep disturbance [24].

### 3.5. Use of Telehealth and Digital Technology

As suggested above, utilizing telehealth and digital technology may benefit both patients and their providers. On the provider side, it can facilitate brief screening and interventions that could be integrated into routine patient visits. For patients, digital tools add the flexibility of being able to schedule some activities during evenings and weekends when providers may not be available, and it reduces time and costs associated with transportation. Multiple studies within this review used telehealth and digital technology [24,25,26,27,31,32,35,37]. In one case, telehealth and digital tools were combined with in-person sessions [34], while other ACT interventions were fully remote [24,37]. Systematic and narrative reviews included both in-person and remote interventions, but none compared delivery methods for efficacy. This will be an important area for future research.

## 4. Discussion

### 4.1. Summary of Findings

Many cancer survivors experience chronic pain and reduced mental health and quality of life [45]. This narrative review found that ACT shows promise as an adjunctive intervention for chronic cancer-related pain, with improvements reported in psychological flexibility, pain acceptance, emotional distress, quality of life, and pain-related functioning, while effects on pain intensity were less consistent. However, the evidence is limited by heterogeneous study designs, small samples, and relatively few oncology-specific randomized trials. Importantly, no included study directly evaluated ACT for cancer-related pain in primary care. Thus, the primary care considerations presented below should be viewed as evidence-informed extrapolations rather than evidence-based clinical recommendations.

### 4.2. Pain and Suffering in the Context of Cancer Survivorship

Suffering has been defined as the “sum total of the emotional, mental, behavioral, social, occupational, and lifestyle effects of pain” (p. 166, [46]). Within the context of cancer survivorship, suffering can also refer to the existential pain that may result from the diagnosis of a life-limiting condition [12]. Therefore, suffering may not only drive chronic pain but exacerbate it, as reflected in Dame Cicely Saunders’ ‘total pain’ concept [3,4].

On a similar note, Flor and Turk describe chronic pain in the context of a learning process resulting from unconditioned stimuli that lead to physiological, psychological, and biochemical responses [47]. Over time, the person’s ability to cope with the stressors driving pain breaks down, resulting in fear of pain and avoidance behaviors [47]. The Fear-Avoidance model of chronic pain, as first proposed by Vlaeyen and colleagues, posits that individuals experiencing acute pain who subsequently develop fear of pain develop pain avoidance behaviors, and with that, chronic pain [48]. At this point, it becomes difficult to disentangle pain from the emotional suffering which drives and perpetuates it. As Hayes et al. point out, experiential avoidance (in this case, strategies to avoid pain) has an effect that is opposite to what is intended [12]. That is, they lead to maladaptive and defensive behaviors that interfere with pleasurable events in the individual’s life. Treating the physical symptoms of pain therefore becomes ineffective unless the psychological context within which pain is occurring is addressed as well.

As survivorship care increasingly shifts toward primary care, clinicians are often asked to manage persistent pain within brief office visits and with limited access to behavioral health specialists. These realities are particularly pronounced in many low- and middle-income countries, where specialty pain and mental health services remain scarce [41,42]. Although ACT has not yet been directly evaluated for cancer-related pain in primary care, its emphasis on brief, values-oriented behavioral strategies, psychological flexibility, and adaptability across delivery formats suggests that it may be well suited for integration into resource-constrained healthcare settings.

### 4.3. Potential Clinical Principles for Primary Care

As suggested above, the following principles should be viewed as evidence-informed extrapolations rather than evidence-based clinical recommendations. These considerations synthesize findings from oncology studies, broader chronic pain literature, and emerging evidence regarding ACT in integrated primary care and digital health settings. Their purpose is to illustrate how the existing evidence may inform clinical practice in resource-constrained environments while highlighting areas requiring future investigation. Table 3 complements this discussion by providing illustrative examples of how these principles might be operationalized within routine primary care. The examples are intended to demonstrate potential applications of ACT-informed care across multidisciplinary primary care teams and should not be interpreted as standardized protocols or practice guidelines.

### 4.4. Integrated Medical Management

Cancer-related pain is a multidimensional condition requiring comprehensive management that often includes pharmacologic therapy, rehabilitation, and psychosocial support [2]. Consistent with the concept of “total pain,” ACT is not intended to replace medical evaluation or analgesic treatment, but rather to complement existing care by addressing psychological flexibility, pain acceptance, and engagement in meaningful activities despite persistent symptoms. Across the reviewed literature, improvements in pain-related functioning, emotional distress, and quality of life were more consistently demonstrated than reductions in pain intensity, suggesting that ACT may provide added value as part of a multidisciplinary approach to chronic cancer pain rather than as a stand-alone intervention.

### 4.5. Brief ACT-Informed Conversations

Although traditional ACT is delivered as a structured psychotherapy, several core concepts—including values clarification, present-moment awareness, acceptance of difficult experiences, and encouragement of values-consistent behavior—may be incorporated into brief clinical encounters. Primary care clinicians are unlikely to deliver comprehensive ACT protocols during routine office visits. However, brief ACT-informed conversations may reinforce adaptive coping, normalize emotional responses to chronic illness, and encourage continued engagement in meaningful daily activities. Such an approach is consistent with the realities of primary care, where clinicians frequently manage complex chronic conditions within limited consultation times. An ACT-informed conversation in primary care should be brief, conversational, and context-sensitive, with attention to the patient’s current life circumstances, barriers, supports, and values rather than reliance on a one-time checklist-style assessment [49].

### 4.6. Function and Values over Pain Intensity

The literature reviewed suggests that ACT most consistently improves pain interference, functioning, psychological flexibility, pain acceptance, and quality of life, whereas improvements in pain intensity are generally smaller and less consistent. This distinction has practical implications for primary care, where successful management of chronic cancer pain may be better reflected by improvements in daily functioning, participation in valued activities, and overall quality of life than by complete elimination of pain. Incorporating values-based goals into routine follow-up may therefore provide a patient-centered framework for monitoring progress alongside traditional assessments of symptom severity.

### 4.7. Digital and Hybrid Delivery

Many cancer survivors experience barriers to accessing behavioral health services, including transportation challenges, treatment-related fatigue, workforce shortages, and geographic distance from specialty centers [27]. Emerging evidence suggests that telehealth, digitally delivered ACT, and hybrid models combining digital resources including smartphone apps [50] with clinician support may expand access while remaining compatible with the realities of primary care practice. Although direct evidence for these delivery models in cancer-related pain remains limited, their flexibility may facilitate broader implementation, particularly in underserved communities and low-resource healthcare systems.

### 4.8. Behavioral Health Referral

ACT-informed primary care should not be viewed as a substitute for specialty behavioral health services. Patients experiencing severe psychological distress, complex trauma, significant psychiatric comorbidity, uncontrolled pain, or substantial functional impairment may require referral to behavioral health clinicians, pain specialists, or multidisciplinary survivorship programs. Within this model, the role of primary care is to recognize psychosocial contributors to pain, introduce basic ACT-informed principles when appropriate, coordinate care, and facilitate timely referral for patients requiring more intensive intervention.

### 4.9. Illustrative Clinical Scenario

A 58-year-old breast cancer survivor presents to her primary care physician with chemotherapy-induced peripheral neuropathy despite pharmacologic management with duloxetine [51]. Although her pain severity has remained relatively unchanged over the preceding six months, she reports withdrawing from activities she previously enjoyed, including gardening and caring for her grandchildren, because of persistent problems with insomnia, anxiety, and physical discomfort. During a routine 20 min follow-up visit, the clinician briefly explores how pain has affected her daily functioning and asks which activities remain most important to her. Together, they identify spending time with family as a core personal value and establish a realistic goal of resuming short periods of gardening several times during the coming week, accepting that some discomfort may be present while avoiding unnecessary activity restriction. The clinician reinforces that improving quality of life and maintaining valued activities are important treatment goals alongside medication management, recommends an evidence-based online ACT program for additional self-management support, and schedules follow-up to review progress while continuing her existing pharmacologic regimen. If symptoms of depression, severe anxiety, or worsening functional impairment emerge, referral to a behavioral health specialist with expertise in chronic pain management would be appropriate.

## 5. Limitations

Several limitations should be considered when interpreting this narrative review. Although the search strategy and inclusion/exclusion criteria were developed by a team, the literature screening was conducted by a single author, creating the possibility that relevant studies were overlooked. Second, the review was limited to peer-reviewed, English-language publications and excluded gray literature, including dissertations, conference proceedings, and non-peer-reviewed reports. This restriction may be particularly important for LMICs and other resource-constrained settings where relevant ACT interventions may be less likely to appear in indexed peer-reviewed journals. The included literature was also heterogeneous with respect to study design, cancer population, intervention format, and outcome measurement, limiting direct comparisons across studies. In addition, the review included both primary studies and secondary (evidence) reviews, and the relatively small number of experimental studies limits the strength of conclusions regarding effectiveness. The absence of a formal risk-of-bias or methodological quality assessment limits conclusions regarding the certainty of the evidence. This limitation is particularly important given the small samples and heterogeneous designs represented in the literature, and findings should therefore be interpreted as preliminary. Finally, none of the included studies directly evaluated ACT for cancer-related chronic pain in primary care; therefore, the proposed clinical considerations represent evidence-informed extrapolations rather than empirically validated primary-care recommendations.

## 6. Future Research

Future research should move beyond efficacy studies conducted in specialty settings and evaluate implementation of ACT within routine primary care. Pragmatic trials are needed to determine whether brief ACT-informed interventions can be feasibly integrated into multidisciplinary primary care teams and delivered through digital or hybrid models, particularly in resource-constrained settings. In addition to pain intensity, future studies should evaluate outcomes such as pain interference, functional status, quality of life, psychological flexibility, implementation outcomes, and cost-effectiveness. Such research will help determine whether ACT can become a practical component of comprehensive cancer survivorship care in primary care.

## 7. Conclusions

ACT shows promise as an adjunctive approach to chronic cancer-related pain, particularly for improving pain acceptance, emotional distress, functioning, and quality of life. However, no published studies have evaluated the use of ACT in primary care for addressing these symptoms among cancer survivors. Rather than replacing medical management, ACT may complement treatment by addressing psychological and functional dimensions of cancer pain. Brief ACT-informed conversations, interdisciplinary care, and values-based approaches may be particularly relevant to primary care, where time and resources are scarce. Integration of ACT will require feasibility studies to address administration and implementation issues, including provider training, utilization of digital resources, acceptability among patients, and, potentially, the utilization of non-licensed providers (e.g., community health workers) to augment existing staff resources.

## Figures and Tables

**Table 1:** Studies utilizing ACT for cancer pain: Country of origin, participant demographics, type and stage of cancer.

Authors	Country of Origin	Number of Participants	Age/Gender	Type of Cancer	Cancer Stage
Andreevich et al., 2023 [23]	Iraq	*n* = 150	34% < age 50, 66% age 50+ 63% male	Stomach	Not specified
Burns et al., 2023 [24]	United States	*n* = 40 *	*μ* = 58 55% female	Gastrointestinal	III, IV
Fashler et al., 2018 [25]	Canada	*n* = 556	N/A	Breast, ovarian, colorectal, mixed	N/A
Feng et al., 2021 [26]	China	*n* = 843	*μ* > 50 % Female: 40–100	Breast, colorectal, myeloproliferative neoplasm, hematologic, lung	N/A
Garcia-Torres et al., 2024 [27]	Spain	*n* = 659	*μ* = 56 % Female: 50–100	Not specified	I-III
Ghorbani et al., 2021 [28]	Iran	*n* = 40	*μ* = 46 100% Female	Breast	N/A
Graham et al., 2016 [29]	United Kingdom	*n* = 448 (275 with cancer)	N/A	Ovarian, colorectal, mixed	N/A
Hadlandsmyth et al., 2019 [30]	United States	*n* = 54	*μ* = 53	Breast	N/A
Kumar et al., 2026 [31]	Australia	Not disclosed	N/A	Breast, prostate, melanomas, intestinal, colorectal, ovarian, nasopharyngeal, mixed	N/A
Li et al., 2021 [32]	China	*n* = 261	*μ* = 62, gender not specified	Ovarian, lung, breast, colon, myeloma, prostate, bowel, non-Hodgkin lymphoma, pancreas, uterus, leukemia	III, IV
Lim & Ahmad, 2026 [33]	Malaysia	*n* = 12	*μ* = 55 100% female	Breast	N/A
Malins et al., 2020 [34]	United Kingdom	*n* = 99 (36 with cancer)	*μ* = 55 69% female	Breast, GI, hematologic, head and neck, gynecologic, urologic, lung, liver	N/A
Mathew et al., 2021 [35]	India and United States	*n* = 537	*μ* = 51 75–100% female	Breast, brain, testicular, ovarian, mixed	N/A
Van de Graaf et al., 2023 [36]	Netherlands	*n* = 12	*μ* = 64 66% female	Bladder, breast, colorectal, ovarian, multiple myeloma, lung	N/A
Van de Graaf et al., 2025 [37]	Netherlands	*n* = 12	*μ* = 64 66% female	Bladder, breast, colorectal, ovarian, multiple myeloma, lung	N/A

* Study included patients and caregivers. Demographics are for patients only.

**Table 2:** ACT for cancer pain study designs, types of pain studied, and study results.

Authors	Study Design	Types of Pain Studied	Study Results
Andreevich et al., 2023 [23]	Semi-experimental pretest, posttest with control group.	Psychological constructs (anxiety, QoL) related to cancer pain.	ACT enabled patients to accept the physical sensations and anxiety associated with stomach cancer and improve QoL. Beck Anxiety Inventory (*p* < 0.001). SF-36 QoL Questionnaire (*p* < 0.001).
Burns et al., 2023 [24]	Secondary analysis of a pilot RCT using telephone-based dyadic ACT.	Psychological and physical pain severity and interference.	Compared to the control (education support), patients and caregivers receiving ACT experienced moderate reductions in physical pain severity/interference (effect size 0.47) and moderate reductions in depression (effect size 0.42).
Fashler et al., 2018 [25]	Narrative review of 1 case study, 3 pre-post cohort studies, and 2 RCTs.	Psychological and physical pain, emotional distress and traumatic responses.	ACT interventions reported by authors to have significantly improved QoL and psychological flexibility with reductions in distress, emotional disturbances, physical pain, and traumatic responses. Statistics not reported.
Feng et al., 2021 [26]	Systematic review and meta-analysis of 10 RCTs, including 2 utilizing ACT delivered telephonically.	Pain intensity and pain interference.	MBIs including ACT resulted in significant reductions in pain intensity (SMD −0.19–0.20, 95% CI) but not pain interference (−0.24–0.10 95% CI).
Garcia-Torres et al., 2024 [27]	Systematic review of 7 studies having at least 2 of the 6 main ACT components delivered electronically.	Emotional distress, pain interference and intensity.	One study using VAS found significant reductions in pain (*p* < 0.001), while a second study did not find significant improvements in pain intensity/interference (measured with PROMIS-29). Studies showed significant reductions in stress, anxiety, depression (*p* < 0.05), and emotional distress.
Ghorbani et al., 2021 [28]	Pretest-and-posttest clinical trial.	Depression and pain acceptance.	Significant improvements in depression, pain acceptance and psychological flexibility (*p* < 0.05) in the treatment compared to the control group.
Graham et al., 2016 [29]	Systematic review of 18 RCTs using ACT to reduce distress and increase symptom control.	Emotional distress related to cancer pain.	Three studies utilizing ACT for patients with colorectal, late-stage ovarian, and unspecified cancers found significant reductions in emotional distress (as measured by mean ES) following ACT interventions.
Hadlandsmyth et al., 2019 [30]	Pilot RCT utilizing a single session of ACT, with participants interviewed 3 months post-surgery.	Post-surgical pain and anxiety.	Small positive effects for use of ACT to address post-surgical pain (Phi = 0.08) and anxiety (Phi = 0.16) among women with breast cancer.
Kumar et al., 2026 [31]	Scoping review of 84 original research articles, including quantitative and mixed methods	Emotional distress (worry, depression, insomnia).	Significant improvements in cognitive behavioral interventions including ACT for worry, depression, and sleep efficiency (statistics not reported).
Li et al., 2021 [32]	Systematic review of 6 studies including 5 RCTs and one pretest/posttest design.	Emotional distress, depression, and physical pain.	ACT was effective in improving psychological distress, anxiety, and insomnia (significant, *p* = 0.001), with non-significant changes in fatigue and pain.
Lim & Ahmad, 2026 [33]	Qualitative clinical trial with 12 participants in a group intervention (no control), and 9 completing the full intervention.	Pain perception.	In patient interviews, participants found the intervention useful and culturally competent, with meaningful social connections. ACT contributed to functional adjustments and pain acceptance. However, participants also noted barriers with regard to transportation and scheduling that might be addressed with remote delivery and/or shorter sessions.
Malins et al., 2020 [34]	Non-randomized cohort study with control	Chronic pain, nausea, fatigue and insomnia, and pain acceptance.	An MI telephone intervention using reflections and summaries to reduce dropout in cognitive behavioral therapies including ACT for cancer and chronic pain. MI significantly increased adherence (d = 0.84) and reduced dropout compared to the control group, resulting in increased pain acceptance (*p* = 0.038) in the experimental group.
Mathew et al., 2021 [35]	Systematic review of 13 studies, reporting on 537 cancer survivors	Physical pain and vitality, anxiety, depression, rumination, and stress biomarkers.	ACT significantly reduced anxiety and depression, and improved quality of life (statistics not reported), while physical pain and insomnia were understudied.
Van de Graaf et al., 2023 [36]	Semi-structured interviews with patients, healthcare professionals, and e-health experts to identify online intervention needs.	Peripheral neuropathy.	Overall, 10 themes were identified in patient interviews: psychosocial aspects, overall intervention need, exercises, content, intervention development, usability, guidance, peer support, comorbidities and implementation.
Van de Graaf et al., 2025 [37]	Qualitative study including 12 patients with CIPN who participated in the *Embrace Pain* RCT.	Barriers and facilitators to engagement in the online ACT self-help protocol.	Barriers included program schedule, lack of guidance, irrelevance, mindfulness exercises, usability, and missing content. Facilitators included usability, recognition, positive self-management, program schedule, symptom management, relevance, guidance, experiential exercises, mindfulness exercises, and value-based living.

**Table 3:** ACT-informed clinical considerations for primary care management of chronic cancer-related pain.

Clinical Consideration	Rationale from Current Evidence	Example in Primary Care
Complement medical management with ACT-informed care	ACT consistently improves pain-related functioning, psychological flexibility, and quality of life, but should complement—not replace—medical evaluation and pharmacologic management.	During rooming, a nurse asks, “What activities that are important to you has pain prevented you from doing this week?” The response becomes part of the clinical discussion alongside pain severity and medication effectiveness.
Focus on meaningful functioning in addition to pain intensity	Improvements in pain interference, daily functioning, and quality of life are more consistently observed than reductions in pain intensity.	During follow-up, the clinician documents both pain intensity and a patient-selected functional goal (e.g., walking a grandchild to school, attending religious services, gardening, or preparing meals).
Incorporate brief ACT-informed conversations	ACT principles may be adapted to brief encounters without delivering formal psychotherapy.	During a 15 min visit, the physician acknowledges that pain may persist while exploring one personally meaningful activity the patient wishes to regain and encourages one small values-consistent goal before the next visit.
Use the interdisciplinary care team	Primary care is increasingly team-based, allowing behavioral support to extend beyond the physician encounter.	A behavioral health consultant, nurse, community health worker, or trained frontline worker follows up between visits to reinforce value-driven, functional goals, assess barriers, and encourage continued engagement in valued activities.
Leverage digital and hybrid delivery models	Emerging evidence suggests digital and hybrid ACT models may improve access, particularly where behavioral health resources are limited.	The clinician recommends an evidence-based online ACT program or smartphone application (e.g., ACT Coach, ACT Companion) and briefly reviews progress during routine follow-up visits while continuing standard medical management.
Recognize when referral is appropriate	Patients with severe psychological distress or complex pain presentations require multidisciplinary management.	Patients demonstrating worsening depression, severe anxiety, trauma-related symptoms, uncontrolled pain, or substantial functional decline are referred to behavioral health, pain medicine, oncology, or palliative care while primary care continues longitudinal medical management.

## Data Availability

No new data were created or analyzed in this study. Data sharing is not applicable to this article.
